# Digital Twin Model of Electric Drives Empowered by EKF

**DOI:** 10.3390/s23042006

**Published:** 2023-02-10

**Authors:** Mohsen Ebadpour, Mohammad (Behdad) Jamshidi, Jakub Talla, Hamed Hashemi-Dezaki, Zdeněk Peroutka

**Affiliations:** 1 Research and Innovation Center for Electrical Engineering (RICE), Faculty of Electrical Engineering, University of West Bohemia (UWB), 30100 Pilsen, Czech Republic; 2 Faculty of Electrical Engineering, University of West Bohemia (UWB), 30100 Pilsen, Czech Republic

**Keywords:** sensorless control, Extended Kalman Filter (EKF), digital twin, induction motor (IM), state estimation, Metaverse

## Abstract

Digital twins, a product of new-generation information technology development, allows the physical world to be transformed into a virtual digital space and provide technical support for creating a Metaverse. A key factor in the success of Industry 4.0, the fourth industrial revolution, is the integration of cyber–physical systems into machinery to enable connectivity. The digital twin is a promising solution for addressing the challenges of digitally implementing models and smart manufacturing, as it has been successfully applied for many different infrastructures. Using a digital twin for future electric drive applications can help analyze the interaction and effects between the fast-switching inverter and the electric machine, as well as the system’s overall behavior. In this respect, this paper proposes using an Extended Kalman Filter (EKF) digital twin model to accurately estimate the states of a speed sensorless rotor field-oriented controlled induction motor (IM) drive. The accuracy of the state estimation using the EKF depends heavily on the input voltages, which are typically supplied by the inverter. In contrast to previous research that used a low-precision ideal inverter model, this study employs a high-performance EKF observer based on a practical model of the inverter that takes into account the dead-time effects and voltage drops of switching devices. To demonstrate the effectiveness of the EKF digital twinning on the IM drive system, simulations were run using the MATLAB/Simulink software (R2022a), and results are compared with a set of actual data coming from a 4 kW three-phase IM as a physical entity.

## 1. Introduction

Digital manufacturing has been beneficial for modern industries because of the advances in communication and information technology. This has been made possible through the use of digital twin (DT) models and process development simulations [1,2,3]. The digital twin model, which is a system that can simulate the physical twin and is made up of multiple probabilistic phases, has been developed since around 2000. It is a formal concept that allows for the creation of a feasible model [4]. A digital twin needs a platform to create a digital simulation of a real-world process, as well as to perform simulations of electric drive systems. The Metaverse is an excellent virtual platform for digital twin models because it combines digital and physical environments through the use of communication technologies, web infrastructures, and the Internet of Things. Many researchers have been interested in various platforms, but the Metaverse, with its ability to allow users to work in a virtual world through avatars, has shown the importance of conducting research [5,6]. In a study [7], the instability in flexible fixturing systems caused by incomplete constraints and weak rigidity has been addressed. This way, a digital twin-based approach to generate reconfigurable fixturing schemes, using a hidden Markov model to account for time-varying system stiffness and coupling effects, was proposed. The optimization process was guided by local information from front-running simulations. The approach was demonstrated through trimming experiments on a large compliant workpiece using a reconfigurable fixturing system developed by the authors.

The practical implementation of electric drives has been difficult for manufacturers and researchers due to the high cost of installation and the time-consuming nature of the process. This has made digital twinning a useful application in these situations. Three-phase induction motors (IMs) have been widely used in a variety of industrial projects, such as electric vehicle propulsion drives, railway traction, and aerospace industries, due to their simple and robust construction, low maintenance, and cost-effectiveness [8]. Nevertheless, controlling Ims can be complex due to their highly nonlinear model, which has an unknown load profile and time-varying parameters. This requires a thorough understanding of rotor flux and position/speed to be effective. In addition, speed sensorless IM drives have several advantages, including low cost, reduced complexity, and improved reliability [9,10]. Digital twins for IM drive systems are not commonly used for drive performance monitoring, health diagnostics, state optimization, and risk evaluation at different levels, including the entire system, subsystems, specific components, and others [11].

There have been several approaches to improving the control of Ims, including model-based predictive control (MPC), field-oriented control (FOC), and direct torque control (DTC). There have been numerous reports that have combined the benefits of IM drives with sensorless control techniques to create low-cost, high-performance actuators [12,13]. In the literature, there have been a variety of approaches proposed for sensorless control of IM drives, but many challenges and uncertainties remain. To address these issues, several model-based estimation techniques have been suggested in the literature, including full-order estimators [14], sliding-mode observers [8], nonlinear Kalman filters, extended Luenberger observers (ELO), and model reference adaptive systems (MRAS) [15].

Nonlinear Kalman filter-based observers, the most commonly used algorithm, provide a probabilistic view of state estimation problems because they rely on the measurement and system noise covariance in the estimation process [16]. In recent years, various nonlinear Kalman filter algorithms such as the Extended Kalman filter (EKF) [17], unscented Kalman filter (UKF) [18], and cubature Kalman filter (CKF) [19] have been applied to the state/parameter estimation of Ims. The EKF method, which is a linearized version of the traditional Kalman filter, serves as an optimal state estimator for actual values of Ims such as rotor flux, stator currents, speed, and parameters based on the system Jacobian matrix. Additionally, the computational requirements of the EKF, which are largely influenced by the size of the motor states, are lower than those of the other mentioned nonlinear Kalman filters. These beneficial features make the EKF estimator more general and universal compared to most other observers.

The integration of the Kalman filters with a virtual asset of the real system, called DT, can offer real-time process evaluation. Considering of such data in actual system operation can enhance control performance with improved productivity. Several examples in the literature present use of the EKF algorithm for DT purposes in different aspects and applications. DT of the backer’s yeast batch cultivation system is analyzed in [20] based on the linear discrete Kalman filter, and its nonlinear variants such as EKF and UKF are used to predict the bioprocess variables employed for optimization and control of the real entity. Various aspects of Li-ion battery prognostics and lifespan monitoring are reviewed in [21] using the DT technology based on the EKF algorithm for estimation of state-of-charge, capacity, current/voltage, and remaining-useful-life. It concludes that the combination of DT with an EKF observer can prevent the battery from having severe failure, optimize its maintenance schedules, and conduct health monitoring with a focus on fault identification and correction. In study [22], the sparse detection of nonlinear dynamics challenges is integrated with the EKF algorithm to automatically recognize the time-variant DT models for online system monitoring. The robustness of the algorithm is validated through a simulation model based on Lorenz process and an industrial diesel hydrotreating case. A research work in [23] presented technical features of merging machinery with a physics-based DTs based on EKF methods for providing successful implementation information. In this regard, two industrial processes were analyzed to demonstrate the methodology; the first is a mobile log crane, which is applicable for the heavy mobile machinery, and the second process concerned a rotating electric machine in case of unexpected failures.

To obtain a reliable state estimate of an IM using the EKF, accurate information on stator phase voltages is essential. Many previous studies have used an ideal inverter model in the IM drive with low-precision stator voltage knowledge as input to the EKF algorithm. However, the switching devices of an inverter are not ideal in practice, as they have small dead times, turn-on and -off times, and voltage drops in each switching interval to prevent shoot-through on the DC link. Dead time creates a nonlinear converter feature that distorts the stator voltage. In other words, it produces a set of low-order harmonics in the stator voltage of the drive inverter, leading to additional losses, particularly at high switching frequencies [24]. To address this distortion and make the model more feasible, the effect of the dead time and voltage drop of inverter switching devices must be taken into account through practical modeling of the inverter. While much research has been conducted on sensorless IM drives based on the EKF using an inaccurate ideal model of the inverter [24,25,26], there is relatively little literature on practical state estimation.

This paper presents a practical digital twin EKF-based sensorless rotor FOC technique for IM drives. Unlike previous studies, the effect of switching devices’ time delays and voltage drops is mathematically analyzed and considered in the estimation process. To evaluate online state estimation, the measurement and system noise covariance matrices are included in the drive model. Accurate values of stator current and rotor flux are obtained in stationary reference frames through a recursive updating process. The rotor position/speed and load torque are also estimated using a precise EKF algorithm. This study is conducted on a 4 kW three-phase IM using MATLAB/Simulink in two operating scenarios, one using an inaccurate ideal model of the inverter and one based on an accurate feasible model. The organization of this manuscript is as follows. In Section 2, the concepts of digital twins and the Metaverse in field of electrical drives are effectively explained. In Section 3, the mathematical model of the drive system, including the IM model, EKF algorithm, and DT model of the three-phase inverter, is presented. Section 4 describes the control strategy based on the rotor FOC method. In Section 5, the simulation results are presented with more numerical analyses, and the final sections contain the discussion, conclusion, and future work.

## 2. Concepts of Digital Twins and the Metaverse in Electrical Drives

### 2.1. Digital Twins and Electrical Drives 

Over the past twelve years, significant research results have emerged, with the concepts of parallel intelligence published in 2004 and digital twins coined in 2010 leading to important implications for the study of Cyber Physical Systems (CPS) and the latter to Cyber Physical Social Systems (CPSS) [27,28,29]. These concepts have provided innovative insights for addressing challenges related to the construction of digital intelligent societies, such as the integration and mining of large amounts of data from Internet of Things devices, the separation between artificial systems and actual systems, and the coordination of diverse, multisource resources. The key difference between digital twins and parallel intelligence lies in their research infrastructure, with the former being related to CPS and CPSS. In addition, digital twins did not initially consider human factors, while parallel intelligence was primarily focused on them [27].

A new method for controlling deformation of thin-walled parts during milling using a digital twin called milling process digital twin was presented in a study [30]. Accordingly, a new reference framework for online optimization and control of milling deformation, which improved the quality of the final product, was introduced. The method was enabled by three key technologies: multidimensional high-fidelity modeling, knowledge-driven low-latency milling deformation simulation, and online optimal control of milling deformation. The authors also presented a prototype system of the proposed method that demonstrated its feasibility in an industrial setting.

In addition, another study [31] has a different perspective and mentioned that in the past, digital modeling techniques such as BIM and data acquisition tools were used for construction and controlling physical objects. However, digital twins (DTs) offer unique features such as bi-directional data exchange and real-time self-management. The need for DTs has increased, particularly after COVID-19, as they can be useful in various industries. The paper aimed to explain the concept of DTs and how they differ from other technologies and systems. The current state of DT development was also reviewed, and suggestions for future research were provided. The focus was on the Smart City, engineering and construction sectors, and the need for DT applications that can provide real-time decision-making, self-operation, and remote supervision capabilities post-COVID-19 [31].

The CPS system, which is made up of the physical and virtual worlds working together, is difficult to fully design from the start. It is meant to provide benefits such as real-time optimization and adaptability [32]. The idea of a digital twin, which is a real-time digital representation of a physical object, is being explored as a way to better understand and control the CPS system.

A digital twin Is a virtual replica of a real-world physical system or product that serves as a digital equivalent for practical purposes such as system simulation, testing, maintenance, and monitoring. This concept is particularly useful for complex systems, where each component affects the overall performance of the system. Materials can also be included in this classification. By using a digital twin in real time and synchronizing it with the corresponding physical system, such as alloys, it is possible to speed up the process of modeling and monitoring the system. Simultaneously running detailed system verification and validation test scenarios on both the digital twin and the physical twin, in this case, a specific alloy, is a practical way to evaluate the effectiveness of the digital twin [33]. The idea behind the digital twin of an electrical drive is to create a digital copy of an electrical drive and use it to improve the real-world performance of that object through simulations and optimization techniques. This is accomplished by constantly updating the digital representation with real data. It is a key aspect of the fourth industrial revolution, specifically in the areas of digitalization and simulation.

### 2.2. Digital Twins of Electrical Drives in the Metaverse

**Figure 1:**
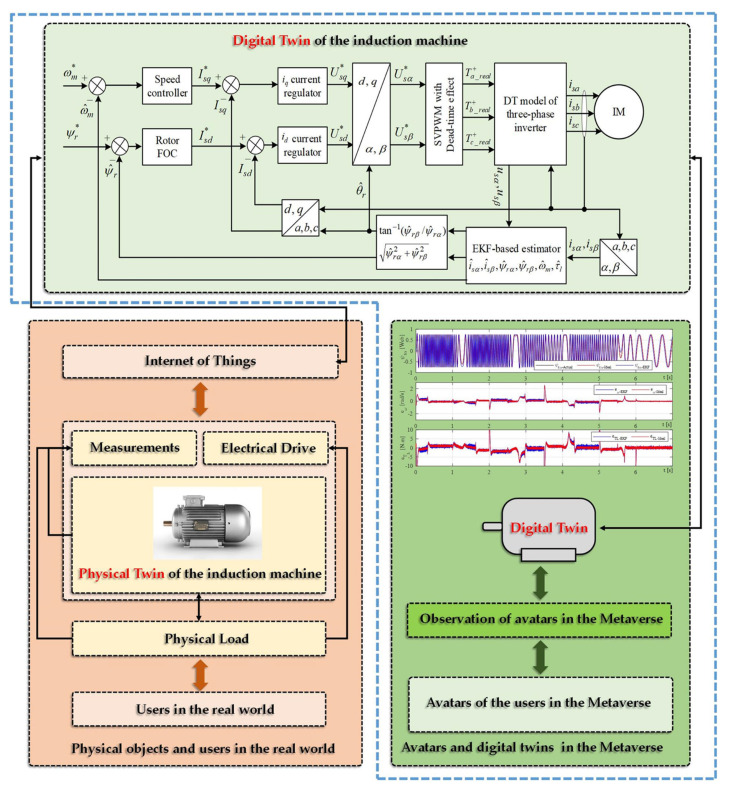
The representation of a physical electrical drive and its digital twin within a Metaverse platform. The use of the term digital twin refers to the concept of creating the digital counterpart, or twin, of the physical electric drive. In this context, the physical electrical drive and its digital twin are existing simultaneously within the Metaverse platform, creating a bridge between the physical and digital realms. The figure highlights the innovative approach of using the Metaverse platform to bring together both the physical and digital aspects of an electrical drive.

To clarify the difference between digital twin and the Metaverse, a digital twin is a virtual replica of an existing physical object or system, whereas the Metaverse is a virtual universe that comprises multiple digital twins and allows for interactions between them. In this study, we focus on the characteristics of the digital twin of an electric drive and also demonstrate some features of the Metaverse, such as value chains, production relationships, and virtual interactions of digital and physical agents. The digital twin of the related electric drive, on the other hand, is used as a virtual representation of its physical twin, and we have collected data from a real system to create it. Figure 1 illustrates the concept of the proposed method.

The use of digital twins of electrical drive technologies across various industries has not been thoroughly studied yet due to the diversity of sub-processes and manufacturing techniques, leading to a vast number of possibilities. The Metaverse, also known as the Internet of 3-D worlds, has recently garnered significant interest from academia and industry. Each virtual subworld, operated by a virtual service provider (VSP), offers a specific type of virtual service. Digital twins, which are digital replicas of physical objects, are crucial enabling technologies. Generally, a DT belongs to the party that develops it and establishes the communication link between the two worlds. However, in an interoperable Metaverse, data-like DTs can be “shared” within the platform, allowing a single set of DTs to be utilized by multiple VSPs. However, the quality of shared DTs may not always be sufficient [34,35,36]. In other words, the Metaverse is a developing technology that creates virtual environments for users to access a wide range of virtual services, while also allowing users to experience immersive interactions with the real world. Digital twins, which represent assets in this virtual world, are crucial in connecting this environment to the actual world.

The potential of data-driven operational support through predictive analysis is limited. A new approach is a model-based simulation of operational behavior, which allows for the simulation of specific physical effects and the monitoring of system behavior even for data that cannot be directly measured. A simulation model that supports plant monitoring is known as a digital twin, which provides additional information about the asset state. Improved knowledge of system behavior increases the availability of the plant and the ability to predict potential faults during operation [37].

The Metaverse can be used to help with the application of these systems by providing a platform for simulation, training, and remote collaboration. One way to use the Metaverse is through simulation. By creating virtual copies of electric drive systems in the Metaverse, engineers and designers can test the syste’s behavior and try out different configurations and control strategies before building the physical system. This can help to identify and solve potential problems before they happen in real life and improve the syste’s performance. Another way to use the Metaverse is through training. By creating virtual training environments in the Metaverse, engineers and maintenance workers can learn how to operate and maintain electric drive systems in a safe and controlled environment. This can help to reduce the risk of accidents and make maintenance operations more efficient. The Metaverse can also be used to make it easier for engineers, researchers, and other experts to work together on electric drive systems. By providing a virtual space where they can share information, work on designs, and conduct experiments, the Metaverse can help speed up the development and deployment of electric drive systems. Data-driven operation support has been a topic of interest for approximately ten years. The effectiveness of methods such as condition-based monitoring or sensor-based fault detection relies on the quantity and placement of sensors. The demand for simulative operation support is relatively new [38].

## 3. Digital Twin Model of the Drive System

The modeling of specific physical processes enables monitoring of the system operation even for information that cannot be directly measured. A simulation model which allows for feasible monitoring is known as a digital twin. In the context of this article, the precise mathematical model to implement digital twins of the electrical drive system includes three parts: the IM, three-phase inverter, and EKF estimator. These parts are explained in detail in the article. In this section, the core task of the digital twin model, which is the state estimation of speed sensorless induction motor drive, is examined. Our approach is to employ a practical EKF algorithm to create digital twins for model-based condition monitoring. This allows for the ability to predict and diagnose potential issues with the system, providing valuable insights for maintenance and operation. In addition, our approach also enables the monitoring of the syste’s performance and identifying areas for improvement.

In the case of an electrical drive system, a digital twin can be created by modeling the different physical processes that take place within the system, such as the operation of the IM, the three-phase inverter, and the control algorithms. This model can then be used to simulate the syste’s performance in real-time. By comparing the simulated results to the actual performance of the physical system, it is possible to identify potential problems and predict future failures. One of the main advantages of using a digital twin for an electrical drive system is the ability to monitor information that cannot be directly measured. For example, a digital twin can be used to estimate the syste’s state, such as the speed and position of the motor, even when there are no sensor data. This is accomplished by using an EKF algorithm which can estimate the syste’s state based on other available information such as current and voltage measurements. In addition, a digital twin of an electrical drive can also be used for condition monitoring, which is the practice of monitoring the condition of a machine or system to detect any signs of wear or degradation. This allows for early detection of potential issues and helps prevent unexpected failures. Additionally, a digital twin can be used to optimize the performance of the system, by identifying areas where energy efficiency can be improved or where the system can be operated more efficiently.

### 3.1. Three-Phase IM Model 

To identify the rotor position of IM and estimate the speed and other states, an accurate motor model along with possible data coming from sensors and simulation results is considered in this study. The sixth-order rotor flux based three-phase IM mathematical model is used for the EKF algorithm in a stationary reference frame (αβ0) to estimate the stator currents ($i_{s\alpha }$, $i_{s\beta }$), rotor fluxes ($\psi_{r\alpha }$, $\psi_{r\beta }$), speed ($\omega_{e}$), and load torque ($\tau_{l}$). To implement the EKF digital twin model on a digital processor or microcontroller platform, it is better to be defined it in a discrete form. The nonlinear discrete IM model can be given as:

(1) $$\left\{ \begin{array}{l} x_{k+1}=f(x_{k},\;u_{k})+w_{k}\\ z_{k}=h(x_{k})+v_{k} \end{array}\right.,$$

The model in (1) can be linearized as follows:

(2) $$\left\{ \begin{array}{l} x_{k+1}=A(x_{k})x_{k}+Bu_{k}+w_{k}\\ z_{k}=Hx_{k}+v_{k} \end{array}\right.,$$

where $x_{k}$ is an (*n* × 1) state vector matrix; $z_{k}$ is an (*m* × 1) current measurement vector matrix; H is the (*m* × *n*) measurement matrix; $f(x_{k},\;u_{k})$ is a known nonlinear function of the state’s transition vector (Jacobian matrix). In this study, it is assumed that the process noise term $w_{k}$ is white and zero mean with an (*n* × *n*) covariance matrix *Q* and that the measurement noise parameter $v_{k}$ is also white and zero mean with an (*m* × *m*) covariance matrix *R*.

The final discrete mathematical model of IM can be obtained as (3) and (4) using the general form in (1) and (2), wherein $u_{s\alpha }$ and $u_{s\beta }$ are the measured stator voltages in stationary reference frame based on the (6). $\omega_{e}$ is the electrical angular speed of the rotor which equals to pole pairs ($p_{p}$) time of the mechanical speed of rotor $\omega_{m}$ (i.e., $\omega_{e}=p_{p}.\omega_{m}$). The motor coefficient matrix $A(x_{k})$ can be expressed as (5).

(3) $$\underset{x_{k+1}}{\underbrace{\left[\begin{array}{l} i_{s\alpha ,k+1}\\ i_{s\beta ,k+1}\\ \psi_{r\alpha ,k+1}\\ \psi_{r\beta ,k+1}\\ \omega_{e,k+1}\\ \tau_{l,k+1} \end{array}\right]}}=A(x_{k})\underset{x_{k}}{\underbrace{\left[\begin{array}{l} i_{s\alpha ,k}\\ i_{s\beta ,k}\\ \psi_{r\alpha ,k}\\ \psi_{r\beta ,k}\\ \omega_{e,k}\\ \tau_{l,k} \end{array}\right]}}+\underset{B}{\underbrace{\left[\begin{array}{l} a_{5}\;0\\ \;0\;\;a_{5}\\ \;0\;\;\;0\\ \;0\;\;\;0\\ \;0\;\;\;0\\ \;0\;\;\;0 \end{array}\right]}}\underset{u_{k}}{\underbrace{\left[\begin{array}{l} u_{s\alpha ,k}\\ u_{s\beta ,k} \end{array}\right]}}+w_{k},$$

(4) $$\underset{z_{k}}{\underbrace{\left[\begin{array}{l} i_{s\alpha ,k+1}\\ i_{s\beta ,k+1} \end{array}\right]}}=\underset{H}{\underbrace{\left[\begin{array}{l} \;1\;0\;0\;0\;0\;0\\ \;0\;1\;0\;0\;0\;0 \end{array}\right]}}x_{k}+v_{k},$$

(5) $$A\left(x_{k}\right)=\left[\begin{array}{cccccc} a_{1}-a_{2}/\tau_{r} & 0 & a_{3}/\tau_{r} & a_{3}\omega_{e,k} & 0 & 0\\ 0 & a_{1}-a_{2}/\tau_{r} & -a_{3}\omega_{e,k} & a_{3}/\tau_{r} & 0 & 0\\ a_{4}/\tau_{r} & 0 & 1-T_{s}/\tau_{r} & -T_{s}\omega_{e,k} & 0 & 0\\ 0 & a_{4}/\tau_{r} & T_{s}\omega_{e,k} & 1-T_{s}/\tau_{r} & 0 & 0\\ -a_{6}\psi_{r\beta ,k} & -a_{6}\psi_{r\beta ,k} & 0 & 0 & 1-a_{7} & -a_{8}\\ 0 & 0 & 0 & 0 & 0 & 1 \end{array}\right],$$

(6) $$\left[\begin{array}{l} u_{s\alpha }\\ u_{s\beta } \end{array}\right]=\left[\begin{array}{l} \;\frac{2}{3}\;-\frac{1}{3}\;-\frac{1}{3}\\ \;0\;\;\;\frac{1}{\sqrt{3}}\;\frac{-1}{\sqrt{3}} \end{array}\right]\left[\begin{array}{l} v_{sa}\\ v_{sb}\\ v_{sc} \end{array}\right],$$

In (5), the parameter $T_{s}$ is the sampling time and the coefficients used in it are listed as follows:

$$\begin{array}{l} a_{0}=\frac{1}{L_{s}L_{r}-L_{m}^{2}},\;a_{1}=1-a_{0}R_{s}L_{r}T_{s},\;a_{2}=a_{0}L_{m}^{2}T_{s},\;a_{3}=a_{0}L_{m}T_{s},\;a_{4}=L_{m}T_{s},\;a_{5}=a_{0}L_{r}T_{s},\;a_{6}=\frac{3p_{p}^{2}L_{m}}{2J_{t}L_{r}}T_{s},\;\\ a_{7}=\frac{B_{t}}{J_{t}}T_{s},\;a_{8}=\frac{p_{p}}{J_{t}}T_{s},\;\tau_{r}=L_{r}/R_{r} \end{array}$$

where $R_{s}$ and $L_{s}$ are the stator resistance and inductance, respectively; $R_{r}$ and $L_{r}$ are the rotor resistance and inductance referred to the stator side, respectively; $L_{m}$ is the mutual inductance; $J_{t}$ is the total moment inertia of the IM and load; $B_{t}$ is the viscous friction coefficient.

### 3.2. Inverter Actual Model 

**Figure 2:**
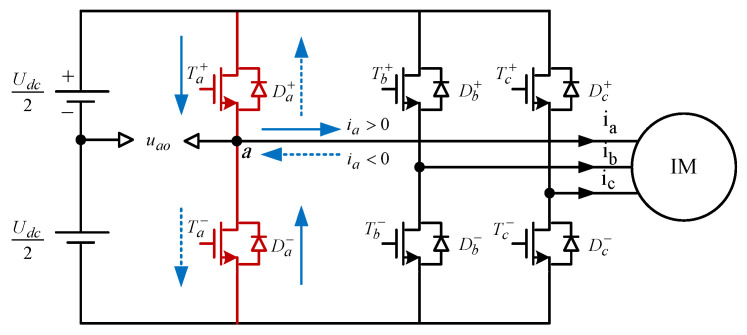
Electric diagram of the three-phase voltage source inverter.

Pulse width modulation (PWM) and space vector pulse width modulation (SVPWM) are two universal switching techniques for the inverters which are feeding the three-phase IMs [39,40,41]. The SVPWM strategy has been widely employed in the IM drive systems due to several merits such as containing small torque ripple, low operation noise, the vast range of DC voltage utilization, and ease. For practical modeling of a three-phase inverter controlled by SVPWM, all switching device′s time delays and voltage drops must be rigorously considered to prevent short-circuit faults that may be caused by simultaneously turning on the two switches of the same bridge leg. Here, a three-phase six-switch voltage source inverter empowered by SVPWM strategy is used for supplying the stator windings of IM, and its A-phase current ($i_{a}$) paths for positive and negative switching intervals are illustrated in Figure 2. The current flowing from the phase to the IM, which is indicated by the blue solid arrow, either through the upper A-phase switch $T_{a}^{+}$ or the lower related diode $D_{a}^{-}$, is determined as a positive phase current, and the current coming from the IM to the inverter leg, which is indicated by the blue dotted arrow, either through the lower A-phase switch $T_{a}^{-}$ or upper diode $D_{a}^{+}$, is determined as a negative A-phase current.

**Figure 3:**
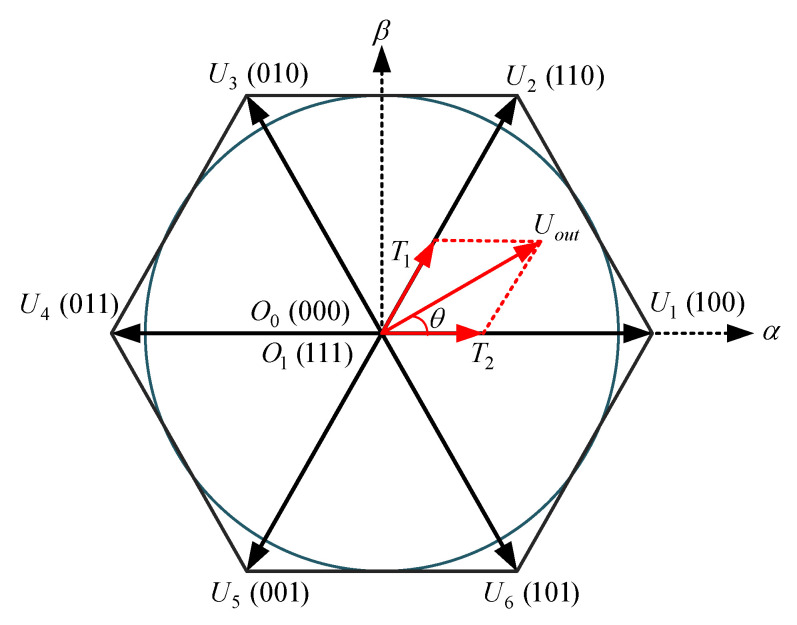
Voltage space vectors based on the SVPWM implemented on TMS320C24x/F24x.

To turn ON and OFF the switching devices of the inverter, the SVPWM technique uses eight switching states with six active sectors to form a PWM signals and shape the output waveform equivalent to a sinusoidal. The voltage space vector diagram of the SVPWM strategy based on the Texas instruments switching patterns (TMS320C24x/F24x code [42]) is depicted in Figure 3, in which $U_{1}-U_{6}$ are the basic active voltage vectors of the six switching sectors with two zero vectors $O_{0}$ and $O_{1}$. $U_{out}$ is the output reference voltage space vector which is synthesized from two adjacent basic voltage vectors (here from $U_{1}$ and $U_{2}$) with interval times of $T_{1}$ and $T_{2}$.

**Figure 4:**
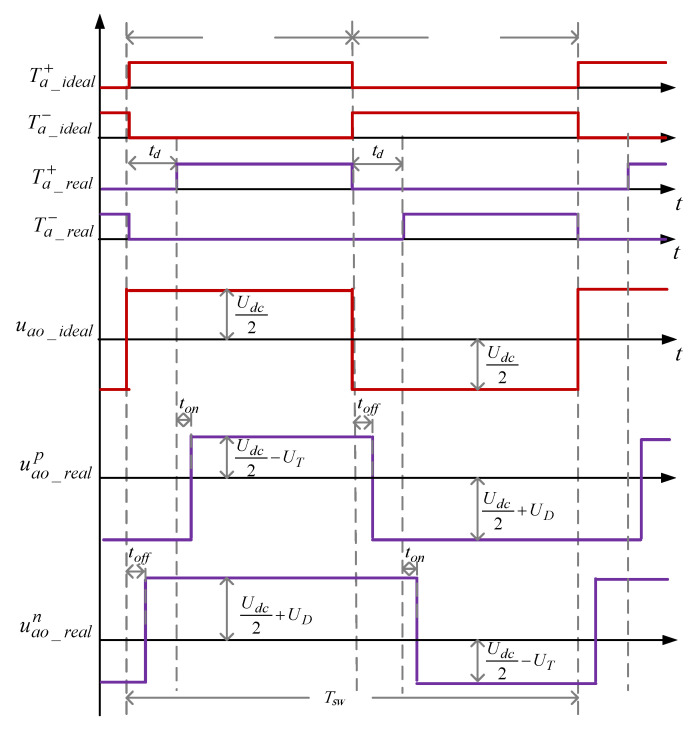
Switching pulses and output voltage for the A-phase during one switching period.

The A-phase corresponding switching time delays analysis is shown in Figure 4. The overall principles of the B and C-phase bridge legs is similar to the A-phase. Figure 4 shows the ideal switching signals for the upper ($T_{a\_ideal}^{+}$) and lower ($T_{a\_ideal}^{-}$) switches of the A-phase inverter leg, the real switching signals based on the dead-time delay $t_{d}$ for the upper ($T_{a\_real}^{+}$) and lower ($T_{a\_real}^{-}$) switches, the ideal voltage waveform of the A-phase leg ($u_{ao\_ideal}$), and the real positive value of A-phase voltage ($u_{ao\_real}^{p}$) and negative ($u_{ao\_real}^{n}$) phase current. Although the dead-time delay in SVPWM setting for practical drives causes distortion in inverter output waveforms and deteriorates the system performance, using the proposed precise EKF algorithm this issue is perfectly addressed. For simplicity of analyzation, it is assumed that the dc-link voltage of the inverter which is denoted by $U_{dc}$ has constant value. The forward voltage drops on the active switches and the diodes, which are denoted by $U_{T}$ and $U_{D}$, respectively, are calculated based on their ON-state resistance characteristics and the absolute value of related phase current as (7).

(7) $$\left\{ \begin{array}{l} U_{T}=r_{T}|i_{a}|+V_{fT}\\ U_{D}=r_{D}|i_{a}|+V_{fD} \end{array}\right.,$$

where $V_{fT}$ and $V_{fD}$ are the inherent forward voltage of the switches and diodes. $T_{on}$ and $T_{off}$ are the ON- and OFF-states times of the upper switches of the inverter, and $t_{on}$ and $t_{off}$ are the finite turn-on and turn-off times of the upper and lower switches, respectively. To prevent a short-circuit in the phase bridge legs of the inverter, the dead-time amount should be chosen as $t_{d}>(t_{on}+t_{off})$. In addition, in practice, the turn-off time $t_{off}$ is a little greater than the switch turn-on time $t_{on}$. It is also necessary to determine $t_{d}>t_{off}$ to avoid simultaneously turning on the upper and lower switches of the bridge legs.

From Figure 4, the output A-phase voltage which is averaged with respect to the midpoint of the dc-link over a switching period $T_{sw}$ for both positive and negative currents can be precisely calculated as:

(8) $${\hat{u}}_{ao\_real}=\left\{ \begin{array}{l} (d-d_{d}-\frac{1}{2})U_{dc}-(d-d_{d})U_{T}-(1-d+d_{d})U_{D}\;,\;\;\;i_{a}>0\\ (d+d_{d}-\frac{1}{2})U_{dc}+(1-d-d_{d})U_{T}+(d+d_{d})U_{D}\;,\;\;\;i_{a}<0 \end{array}\right.,$$

in which d is the ideal duty-cycle of the active switches which is derived from the control module and defined as $d=T_{on}/T_{sw}$, and $d_{d}$ is the overall time delay in a switching period which can be calculated as:

(9) $$d_{d}=(t_{d}+t_{on}-t_{off})/T_{sw},$$

Based on Figure 4 and the inverter digital twin model equation in (8), it is observed that the ON-state time of upper switches in actual operation should be precisely determined as $t_{d}+t_{on}<T_{on}<T_{sw}-t_{d}-t_{on}$; however, in the ideal model, the impact of time delays and voltage drops on switching devices are neglected which results in $0<T_{on}<T_{sw}$; thereafter the output voltage magnitude is changed to as follows:

(10) $${\hat{u}}_{ao\_ideal}=\left\{ \begin{array}{l} (d-\frac{1}{2})U_{dc}\;,\;\;i_{a}>0\\ (d-\frac{1}{2})U_{dc}\;,\;\;i_{a}<0 \end{array}\right.,$$

### 3.3. EKF Algorithm 

In this study, a precise EKF algorithm is used for state estimation of the IM drive system based on the actual model and values of the inverter in comparison with the previous established research which relied on the ideal model. The EKF, as the most preferred nonlinear Kalman filter, provides a stochastic behavior to the state estimation issue, and its success directly depends on the drive system and measurement noises evaluated in the estimation process. In comparison to the other nonlinear estimators, EKF is still a decent choice due to less computational complexity and accurate estimation performance. The operation steps of conventional EKF algorithm based on the three-phase IM model in (3) and (4) can be given as follows:

(1) Initialization

(11) $${\hat{x}}_{0}=E[x_{0}],$$

(12) $$P_{0}=E[(x_{0}-{\hat{x}}_{0}){\left(x_{0}-{\hat{x}}_{0}\right)}^{T}],$$

(2) Linearization

(13) $$F_{k+1|k}=\frac{\partial f(x_{k},\;u_{k})}{\partial x_{k}},$$

(3) Estimation or time updating

(14) $${\hat{x}}_{k}^{-}=f({\hat{x}}_{k-1},\;u_{k-1}),$$

(15) $$P_{k}^{-}=F_{k|k-1}P_{k-1}F_{k|k-1}^{T}+Q,$$

(4) Correction

(16) $$K_{k}=P_{k}^{-}H^{T}{\left[HP_{k}^{-}H^{T}+R\right]}^{-1},$$

(17) $${\hat{x}}_{k}^{+}=(I-K_{k}H){\hat{x}}_{k}^{-}+K_{k}z_{k},$$

(18) $$P_{k}^{+}=(I-K_{k}H)P_{k}^{-},$$

where *F* is the Jacobian matrix to linearize the nonlinear model of IM; $P^{-}$ and $P^{+}$ are the priority and the posterior covariance matrices, respectively; *K* is the Kalman gain; *I* is the identity matrix.

**Figure 5:**
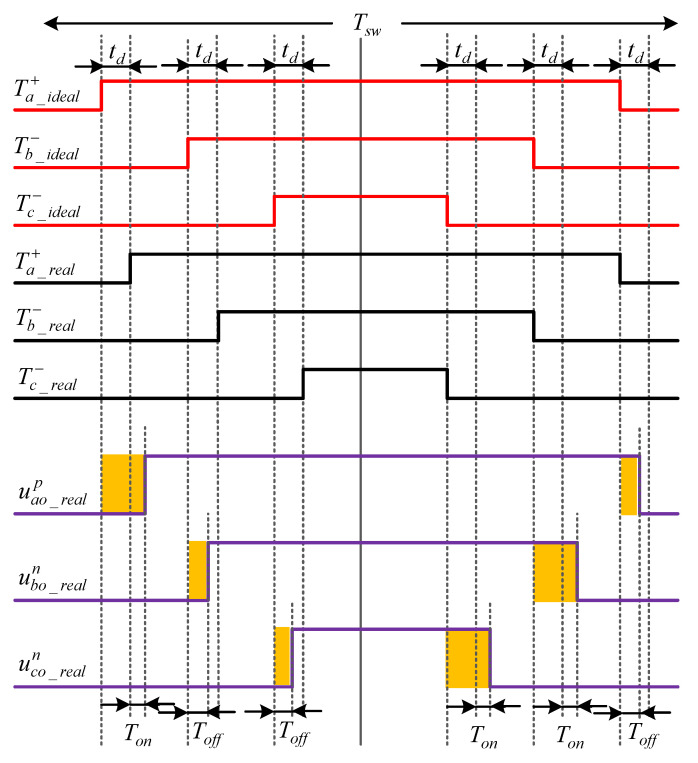
Analysis of time delays effect of the inverter switching devices (depicted in yellow color) during sector I.

From Equations (14) and (15) it can be concluded that the accuracy of EKF-based state estimation strongly depends on IM model, inputs, and parameters. Motor parameters are assumed to be constant over the optimum working modes. The main inputs of the EKF algorithm are stator voltage terms in the stationary reference frame (i.e., $u_{s\alpha }$ and $u_{s\beta }$) which can be originated either from the control system multiplied by dc-link voltage (low precise voltages) or from the voltage source inverter along with precise information of switching devices from SVPWM strategy. Therefore, to practically estimate the IM states, all switching time delays and voltage drops should be considered in input voltage values. To better analyze the proposed precise estimation process, switching patterns and voltage waveforms of the sector I of the SVPWM technique based on Figure 3 are considered as shown in Figure 5.

From Figure 5, the dead-time effect and switching ON and OFF delays impact on the three-phase inverter performance are analyzed when the reference voltage vector $U_{out}$ is located in the sector I wherein $i_{a}>0$, $i_{b}<0$, and $i_{c}<0$. The real positive value of A-phase voltage $u_{ao\_real}^{p}$ is shorter than the ideal value $u_{ao\_ideal}$ by time delay $\Delta T=t_{d}+t_{on}-t_{off}$; for the same reason, the real negative values of B- and C-phase voltages $u_{bo\_real}^{n}$ and $u_{co\_real}^{n}$ are longer than the ideal value by ΔT.

According to Figure 5, when the switching states of A, B, and C phases are compared, the actual switching time of the voltage vector in sector I $U_{1}(100)$ is reduced by 2*Δ*T* in one PWM period (the left side of the positive value and right side of negative value are reduced by Δ*T*). However, the actual switching time of the vector $U_{2}(110)$ in Figure 3 does not change at this time. If the voltage vector difference caused by the dead-time effect is Δ*U*, then the difference between the real voltage vector of the stator $U_{s}$ and the reference voltage vector $U_{out}$ at this time is determined as:

(19) $$\Delta U=-2\times \Delta T\times U_{1},$$

Based on the above analysis, it is clear that there is a sensible difference between the real and ideal voltage which is derived from the three-phase inverter. Hence, the proposed DT model of the inverter which is relied on the actual model can supply precise voltages to the EKF algorithm to practically estimate the speed in sensorless drives.

Here, only the sector I of SVPWM is based on the assumptions of $i_{a}>0$, $i_{b}<0$, and $i_{c}<0$, is analyzed to illustrate that the different positive and negative current polarities can directly affect the results of the stator voltages due to the dead-time effects. As a result, the determination of the polarity and value of the three-phase currents in each sector is a key factor for the analysis of the dead-time effect of the three-phase inverter.

Since during the dead-time intervals there is no information about the three-phase currents due to the off-state of all switches of the inverter bridge leg, the ideal inverter models and conventional EKF algorithms cannot perfectly act to precisely estimate the IM states. The presented DT model of the inverter along with the accurate EKF algorithm fully addressed the mentioned problem by estimating the currents using the actual voltage values based on the above analysis and (8). To estimate the states during the dead-time intervals, the proposed EKF algorithm uses the real current and voltage values from the previous intervals inside the sectors by modifying the voltage in (14) as follows:

(20) $${\hat{x}}_{k}^{-}=f({\hat{x}}_{k-1},\;u_{k-1}^{real}),$$

wherein $u_{k-1}^{real}$ is the actual stator voltage based on the practical DT model of the inverter. 

## 4. Sensorless Control Strategy

**Figure 6:**
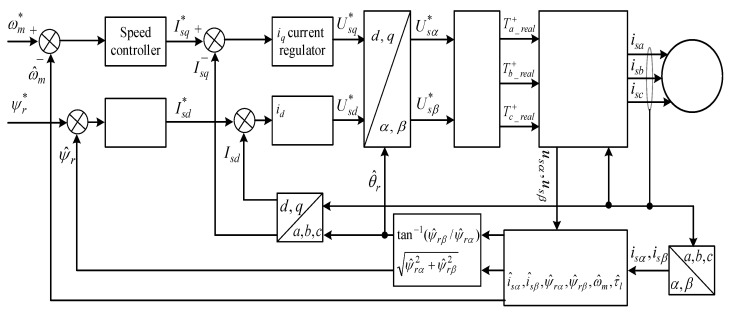
Block diagram of the sensorless control of the IM drives with EKF-based speed estimator.

The overall block diagram of the sensorless rotor FOC strategy based on the precise EKF estimator is shown in Figure 6. The IM drive system consists of two control loops for speed control and rotor flux regulation, SVPWM switching module, DT model of the three-phase inverter, the mathematical model of the IM, the precise EKF estimator, and various sensors related to current and voltage along with the Park and Clark transform blocks. These sensors provide data about different aspects of the IM performance, operational modes, and more. These data are then transferred to a processing system and applied to the digital processors. Once informed with such information, the virtual DT model can be used to run simulations, study performance targets, and produce possible signals/feedbacks which can then be applied back to the original physical asset. As shown in Figure 6, rotor speed and position data are obtained using the estimator instead of employing mechanical sensors, which makes the drive system suitable for cost-effective applications. 

Unlike the conventional current-based control strategy which is affected by variation of rotor resistance results to cause an error in flux value evaluation, for flux amplitude and angle computation the stationary reference frame voltage model is utilized in this paper which is insensitive to the rotor resistance variations. In order to better analyze the control system, the reference angular speed $\omega_{m}^{*}$ is compared to the estimated motor speed ${\hat{\omega }}_{m}$ generated by the EKF estimator, and the speed error is applied to a PI (proportional-integral) speed controller. The output of the speed controller, which is considered as reference electromagnetic torque $\tau_{e}^{*}$, is used to calculate the q-axis reference current $I_{qs}^{*}$ based on (21) wherein ${\hat{\psi }}_{rd}$ is the d-axis estimated rotor flux. $I_{qs}^{*}$ is compared to the measured q-axis current $I_{qs}$, and its error is regulated by the PI controller to generate a voltage control signal $V_{qs}^{c}$ which is utilized to produce q-axis reference voltage $U_{qs}^{*}$ as expressed in (22) in which $\omega_{s}$ is stator field angular speed in terms of rad/s.

The same methodology with the speed control loop is applied to the second loop for the rotor field control, which initially compares the reference rotor flux $\psi_{r}^{*}$ with the estimated flux ${\hat{\psi }}_{r}$ to produce a d-axis reference current $I_{ds}^{*}$ using the PI controller. $I_{ds}^{*}$ is compared to the measured d-axis current $I_{ds}$ results in an error regulated by the PI regulator to generate d-axis voltage control signal $V_{ds}^{c}$ for producing d-axis reference voltage $U_{ds}^{*}$ as computed in (23). From (21) to (23), it is seen that the main control signals strongly depend on the IM parameters which are relayed on the accurate DT model in this study.

(21) $$I_{qs}^{*}=\frac{L_{r}}{p_{p}L_{m}{\hat{\psi }}_{rd}}\tau_{e}^{*},$$

(22) $$U_{qs}^{*}=V_{qs}^{c}+(\frac{\omega_{s}}{a_{0}L_{r}}I_{ds}+\frac{p_{p}L_{m}}{L_{r}}{\hat{\psi }}_{r}{\hat{\omega }}_{m}),$$

(23) $$U_{ds}^{*}=V_{ds}^{c}-(\frac{\omega_{s}}{a_{0}L_{r}}I_{qs}+\frac{L_{m}R_{r}}{L_{r}^{2}}{\hat{\psi }}_{r}),$$

The rotor shaft position which is identical to the rotor field position ${\hat{\theta }}_{r}$ is adopted for measured stator currents transform to dq0 axes based on the Park transformation and the reference voltages ($U_{ds}^{*}$, $U_{qs}^{*}$) to αβ reference voltages ($U_{s\alpha }^{*}$, $U_{s\beta }^{*}$). These voltage signals from the control system are entered into the SVPWM module to apply the dead-time effect and take into account the switching devices′ time delays to generate real switching signals for the upper switches of the inverter, which are denoted by $T_{a\_real}^{+}$, $T_{b\_real}^{+}$, and $T_{c\_real}^{+}$, respectively. Using these practical switching signals and this precise model of the inverter, as explained before, three real stator voltages based on (8) will be produced and applied to the IM. The accurate stator voltages are transformed into two voltages ($u_{s\alpha }$, $u_{s\beta }$) using (6) and utilized as the main inputs along with the measured currents ($i_{s\alpha }$, $i_{s\beta }$) for the EKF estimator.

## 5. Results

To evaluate the proposed EKF estimator performance in different operation modes, the drive system is implemented on MATLAB/Simulink software by employing the DT model of the IM with parameters that are listed in Table 1. At first glance, common ground can be found between DT and hardware-in-the-loop (HIL) simulations, as they both run under real-time simulations; however, the essential difference between DT and HIL implementation is that for the latter one, a software model should be built as an interface with real hardware to evaluate the performance of its controllers. For DT simulation, we need to create only a software model of the drive system that is controlled and then prepare it with inputs and outputs from the controller being tested to identify how well the controller operates and whether it runs what it is supposed to be performing. Although simulations and DTs both use digital models to assess the system’s various processes, a DT is a virtual platform, which makes it considerably richer for study. The difference between digital twins and simulation is largely a matter of scale: while a simulation typically studies one particular process, a DT can run any number of useful simulations to study multiple processes.

Here, for SVPWM modeling, the switching frequency is set to 10 kHz, and $T_{s}$ as sampling time of the algorithm is 1 μs in all simulations. Moreover, the number of time delays $t_{d}$, $t_{on}$ and $t_{off}$ are selected as 4, 1, and 1.5 all in μs, respectively. Fixed-step solver is used in this simulation with ode5 codes. In the simulations, the initial values of all estimated states and parameters are set to zero for both operation scenarios. To avoid the computational burden and simplify the determination of covariance matrices via the trial-and-error method, the *Q*, *R*, and initial *P* matrices in both scenarios are assumed to be the same and considered as constant in diagonal form for the EKF algorithm as follows:

$$\begin{array}{l} Q=diag\{2e-9,\;2e-9,\;4.62e-11,\;4.62e-11,\;4e-9,\;1e-5\}\\ R=diag\{6.7e-3,\;6.7e-3\}\\ P_{0}=diag\{1e-6,\;1e-6,\;1e-6,\;1e-6,\;1e-6,\;1e-6\} \end{array}$$

**Figure 7:**
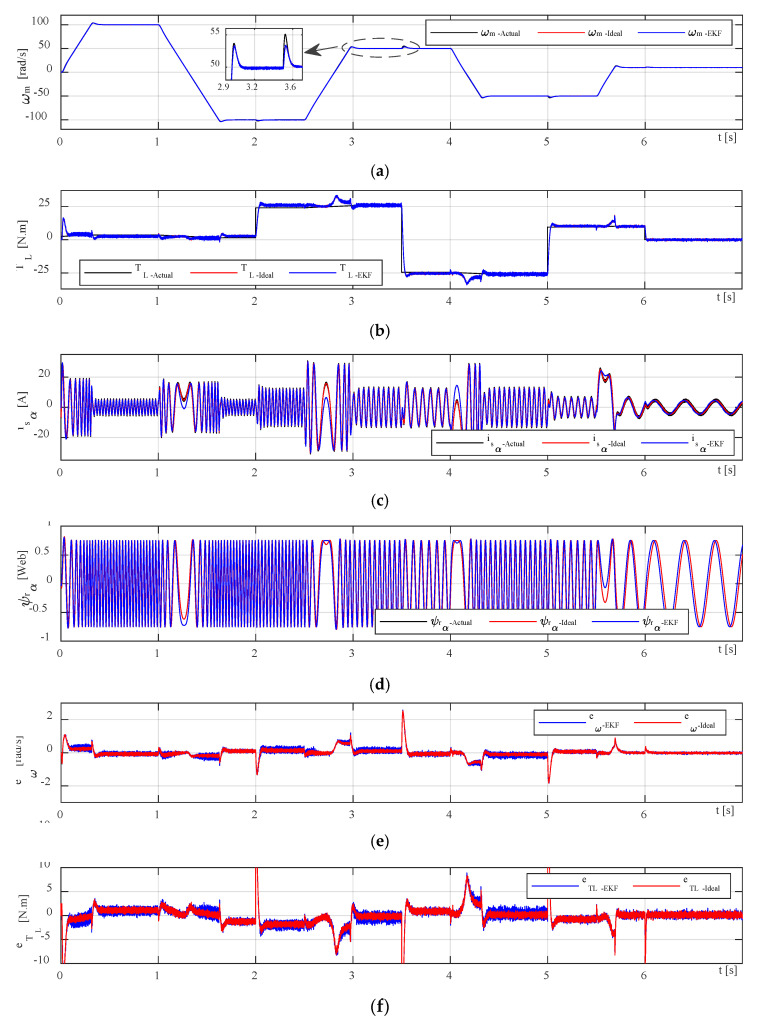
The EKF algorithm estimation results for (**a**) speed, (**b**) load torque, (**c**) current, (**d**) rotor flux, (**e**)speed error, and (**f**) load torque error of the speed-sensorless IM drive.

To verify the performance of the proposed speed-sensorless IM drive system, the simulation studies are carried out in two scenarios. These scenarios are simulated for different speed and load torque profiles based on the values which are presented in Table 2. According to Table 2, the drive system is subjected to positive low (10 rad/s) and high (100 rad/s) speeds as well as negative high speed (−100 rad/s) test along with the variable positive and negative load torque from no-load (0 N·m) to full-load (25 N·m) values. Figure 7 shows the EKF algorithm estimation of the IM drive for both scenarios when the motor is subjected to the nine operation modes based on Table 2. In all figures illustrating the simulation results, the superscripts *Actual*, *Ideal*, and *EKF* indicate the measured states (come from the physical twin which includes the real IM, inverter, and control system), the estimated states by the ideal model of the EKF, and the estimated states by the practical DT model of the EKF, respectively. Moreover, $e_{\left(*\right)}$ denotes the error defined as between the actual and the estimated states. Owing to the DT model of the EKF estimator, the states are estimated without any significant errors in both case studies.

The first scenario deals with an ideal inverter model without considering the switching devices time delays, and voltage drops them. From Figure 7, the IM is loaded to a variable load of minimum −25 N·m to maximum 25 N·m from 0 to 7 s (second subplot) when running with variable speed profile (first subplot) based on Table 2. The performance of the EKF algorithm depends on the predefined noise and measurement covariance matrices and motor parameters. Based on the speed and load torque error results (two bottom subplots), the IM states are precisely estimated with negligible errors in steady states; however, during transients, the estimation errors are increased because of predefined noise matrices, and it is not possible to determine appropriate matrices for all operating modes using the trial-and-error method. The transient errors for the IM speed are about at least 1.5 rad/s and at most 6 rad/s, and for the load torque are about minimum 0.5 N·m and maximum 14 N·m.

The second scenario is simulated based on the feasible practical DT model of the inverter with taking into account all time delays and voltage drops on switches. According to the Figure 7, the performance of the digital twinning EKF algorithm is improved to meet the physical twin model responses with some extra errors and fluctuations in currents and rotor fluxes than the ideal one due to the dead-time effect in steady states operations. Based on the two bottom subplots, the transient errors for the speed are about 2 rad/s to 8 rad/s, and for the load torque are about minimum 1.2 N·m and maximum 16 N·m. It is clear that the error rates in the second scenario with practical model are a little significant due to the time delays of switching devices.

**Figure 8:**
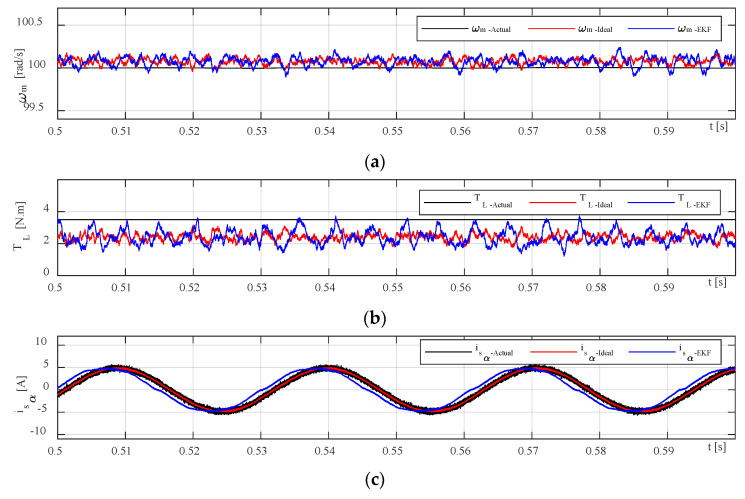

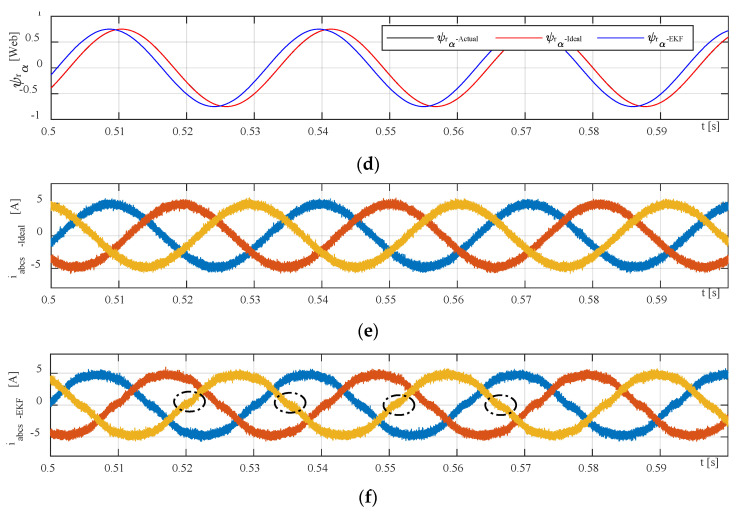
The EKF estimation results in zoomed version for (**a**) speed, (**b**) load torque, (**c**) alpha-term current, (**d**) rotor alpha-term flux, (**e**) actual stator three-phase currents for the ideal model, and (**f**) actual stator three-phase currents for the DT model.

A zoomed part of Figure 7 is illustrated in Figure 8 along with three-phase measured stator currents in both ideal and practical scenarios wherein very small distortions are observed in DT EKF-based current waveforms due to the effect of dead-time and voltage drops of inverter switching devices which are marked with black circles in the bottom subplot of Figure 8. To discriminate the performance of two scenarios, estimated stator currents and rotor flux in stationary frame are more relevant to the point in which the delays caused by dead-time of switches and voltage drops are more considerable.

It should be noted that the measured errors of the system are the reference to control regulation; they might occur in various conditions. Their reasonable fluctuation ranges are set according to knowledge and experience based on experimental records and available measurements/tests. However, the trial-and-error method can be used to set reasonable fluctuation ranges. To ensure that the method is convergent, it is possible to examine the test system under various conditions. Test results showed that the proposed method is convergent.

## 6. Discussion

Computer aided simulations have been effective enough to address design issues and modeling process; however, they are restricted in some cases due to the systems complexity and the high volume of processed data. These types of simulations can be implemented in several in-the-loop simulations tools based on the level of the product generation such as in model-in-the-loop (MIL) form, processor-in-the-loop (PIL) evaluation, software-in-the-loop (SIL) simulation, and HIL. In comparison with the previous simulation tools, DT technology is a decent alternative and a powerful development in area of digital implementation by connecting the virtual and physical entities. DT has already applied in various applications such as industry, automotive, aerospace, healthcare, and medical evaluations. Although electric drive is one of the present DT applications, most investors in this field concentrate on motor design, electric interface, and sensing/measuring. Concerning an electric drive system, it is still a new topic to investigate with DT technology. Among the simulation forms, the HIL simulation is the most similar tool to the digital twin technology. HIL simulation is a remarkable solution for evaluating a drive system or at most an asset performance. It also can be employed in designing or implementing the system and applied for some fault identification. However, DTs can be applied for a component, entity, or the whole drive system due to their major ability to deal with an enormous amounts of various datasets. One of the best implementing solutions in the case of testing electric drive systems might be a combining both DT and HIL technologies.

The basic configuration of our digital twin system illustrated in Figure 1 consists of three main elements: a physical twin (three-phase IM drive system in reak space), a digital representation model in cyberspace, and the interface entity for transferring information to connect all the spaces. The timeline of the simulation running is also shown in Figure 1 with starting from the collecting measured data from physical twin and ending with DT modeling. The overall block diagram of the proposed speed-sensorless IM drive system simulation model is shown in Figure 6, and the estimation performances of EKF algorithm for both ideal and practical operation scenarios are examined for the same references. The resulting control performances and the related errors for the EKF-based version of the proposed drive system are shown in Figure 7 and Figure 8. Moreover, the numerical implementations of the drive system have been run for nine operation modes under two scenarios. Considering the estimation process and control performances which are presented in Figure 6, Figure 7 and Figure 8, the following outcomes can be encapsulated:

- As discussed before, the DT model as a virtual platform makes the practically implementation of the drive system considerably richer for study and improves the performance of the IM drive system that might be applicable for development and research purposes.
- Estimation performances of the EKF algorithm for both ideal and DT models of the inverter at steady-states are close to each other, except for transients in which the DT model contains some distortion in currents due to the switch’s time delays.
- In some cases, challenging low- and high-step changes in speed and load torque are applied to evaluate the proposed drive system’s robustness. Nevertheless, the proposed drive can perfectly address these variations.
- Although the DT model of the EKF algorithm increases the computational burden because of estimating the states inside the sectors in narrow time intervals, its computational load can be acceptable since it improves estimation performance during dead times.
- The main contribution of this work can be practically testing the performance of the speed-sensorless IM drive system based on a digital model in virtual space to use it for further analysis purposes and as a predefined module for future studies with no need to high-cost real test setup along with actual implementation problems and challenges.
- In the case of a technical limitation of the proposed digital drive system, it can be clarified that the most challengeable part of the work is appropriately connecting the physical model data to digital twin model and transferring correct information (care about measurement and system noises) using physical sensors.
- All in all, since DTs offer a lot of functions such as data monitoring, fault detection, optimization, and lifetime prediction, it seems reasonable to employ the DT model of the EKF algorithm instead of the conventional ideal model one for electrical drives especially speed-sensorless drive systems.

In short, although DT technology contains various entities, it is still new and not fully applied for other applications. In case of our proposed digital model of electric drive system, one of the appropriate applications is use of the model for further future research projects with no need for the actual test setup and real physical experiments, which leads to significant reduction in implementation cost. 

## 7. Conclusions

Digital twin is a type of technology that allows the physical world to be represented in a virtual digital space, and it can be used to help build a Metaverse. This technology is a product of the latest advances in information technology. This study involves using a digital twin based on the Extended Kalman Filter (EKF) to simulate and estimate the state of a sensorless electric drive system without a speed sensor, specifically an induction motor (IM), under various operating modes. The Metaverse platform is an appealing option for connecting virtual and physical models through digital simulation using digital twin technology, as compared to other simulation models. The proposed digital twin model significantly improves the performance of the IM drive by providing a practical inverter model, as demonstrated by the estimation results. In contrast to many previous models, this system takes into account the effects of time delays and voltage drops of inverter switches to make it more feasible and reduce current distortions. From the results, it is concluded that the speed and load torque estimation errors, especially in transients for practical modeling based on DT, are a little higher than the ideal model due to taking into account of all time delays of switching devices and voltage drops. 

Future work will concentrate on: (1) applying the proposed DT based EKF observer to other types of electric motors such as permanent magnet synchronous motor and brushless direct current motor; (2) using the parameter estimation along with states estimation to improve the overall performance of the drive system; (3) testing the proposed drive system in different engineering problems, such as robotics, navigation, and data fusion; (4) utilizing inverter dead-time information to accurate evaluation of conduction and switching losses; and (5) using different kinds of nonlinear Kalman Filters such as the CKF or the UKF.

## Figures and Tables

**Table 1:** Main parameters of the sensorless IM drive system.

Three-Phase IM Parameters									
*P* [kW]	*T_L_* [N·m]	*p_p_*	*R_s_* [Ω]	*R_r_* [Ω]	*L_s_* [H]	*L_r_* [H]	*L_m_* [H]	*J_t_* [kg·m^2^]	*B_t_* [N·m/(rad/s)]
4	25	2	1.3177	1.5097	0.1716	0.1716	0.165	0.11	0.01
**Three-phase inverter parameters**									
*r_D_* [mΩ]		*r_T_* [mΩ]			*V_fT_* [V]		*V_fD_* [V]		*V_dc_* [V]
1		1			0.8		0.8		400

**Table 2:** Different operation modes of the proposed IM drive system.

Mode	1	2	3	4	5	6	7	8	9
t [s]	0–1	1–2	2–2.5	2.5–3.5	3.5–4	4–5	5–5.5	5.5–6	6–7
ω*_m_ [rad/s]	100	−100	−100	50	50	−50	−50	10	10
*T_L_* [N·m]	2.5	2.5	25	25	−25	−25	10	10	0

## Data Availability

We will share our research final data link after full revision and publication.
